# Alcohol-related cerebellar degeneration: not all down to toxicity?

**DOI:** 10.1186/s40673-016-0055-1

**Published:** 2016-10-03

**Authors:** Priya D. Shanmugarajah, Nigel Hoggard, Stuart Currie, Daniel P. Aeschlimann, Pascale C. Aeschlimann, Dermot C. Gleeson, Mohammed Karajeh, Nicola Woodroofe, Richard A. Grünewald, Marios Hadjivassiliou

**Affiliations:** 1 Academic Department of Neurosciences, Royal Hallamshire Hospital, and University of Sheffield, Sheffield, UK; 2 Academic Unit of Radiology, University of Sheffield, Sheffield, UK; 3 Matrix Biology & Tissue Repair Research Unit, College of Biomedical and Life Sciences, School of Dentistry, Cardiff University, Cardiff, UK; 4 Academic Department of Hepatology, Royal Hallamshire Hospital, and University of Sheffield, Sheffield, UK; 5 Biomolecular Sciences Research Centre, Sheffield Hallam University, Sheffield, UK

**Keywords:** Alcohol, Ataxia, Cerebellum, Cerebellar degeneration, Transglutaminase

## Abstract

**Background:**

Alcohol-related cerebellar degeneration is one of the commonest acquired forms of cerebellar ataxia. The exact pathogenic mechanisms by which alcohol leads to cerebellar damage remain unknown. Possible autoreactive immune mediated mechanisms have not been explored previously. In this study, we aim to investigate the potential role of alcohol-induced immune mediated cerebellar degeneration.

**Methods:**

Patients with ataxia and a history of alcohol misuse were recruited from the Ataxia and Hepatology tertiary clinics at Sheffield Teaching Hospitals NHS Trust. We determined the pattern of cerebellar involvement both on clinical (SARA score) and imaging (MRI volumetry and MR spectroscopy) parameters. In addition, HLA genotyping, serological markers for gluten-related disorders and serological reactivity on rat cerebellar tissue using indirect immunohistochemistry were assessed.

**Results:**

Thirty-eight patients were included in the study all of whom had ataxia. The gait (97 %), stance (89 %) and heel-shin slide (89 %) were the predominant SARA elements affected. MRI volumetric and spectroscopy techniques demonstrated significant structural, volumetric and functional deficits of the cerebellum with particular involvement of the cerebellar vermis. Circulating anti-gliadin antibodies were detected in 34 % patients vs. 12 % in healthy controls. Antibodies to transglutaminase 6 (TG6) were detected in 39 % of patients and 4 % of healthy control subjects. Using immunohistochemistry, Purkinje cell and/or granular layer reactivity was demonstrated in 71 % of patient sera.

**Conclusions:**

Alcohol induced tissue injury to the CNS leading to cerebellar degeneration may also involve immune mediated mechanisms, including sensitisation to gluten.

**Electronic supplementary material:**

The online version of this article (doi:10.1186/s40673-016-0055-1) contains supplementary material, which is available to authorized users.

## Background

Alcohol misuse is recognized worldwide as a major cause of morbidity and mortality with significant health and economic consequences. The toxic effects of alcohol are diverse. Alcohol-related cerebellar degeneration is one of the commonest causes of acquired cerebellar ataxia. The pathophysiology remains unclear but proposed mechanisms include excitotoxicity, dietary factors, oxidative stress, compromised energy production and cell death [1]. Some argue that the direct toxic effects of alcohol on cerebellar cells is responsible, whilst others report that nutritional factors particularly thiamine deficiency are required to drive the underlying process [2].

Cerebellar atrophy is a recognised result of alcohol-related cerebellar degeneration. The anterior superior cerebellar vermis is predominantly affected [3, 4] with the Purkinje cell, granular and white matter layers being most susceptible [2]. This has been confirmed in large autopsy studies [5, 6]. MRI findings of vermian atrophy, with preferential involvement of the lingula [7], are in keeping with the neuropathological findings.

Metabolic deficits can be demonstrated by magnetic resonance (MR) spectroscopy, with low N-acetyl aspartate:creatine ratio (NAA/Cr) in the cerebellar vermis. MR Spectroscopy can directly detect ethanol by a triple peak at 1.3 ppm [8]. Chronic alcohol use causes a reduction in NAA and choline levels compared to healthy controls; thus MR spectroscopy provides a useful tool for monitoring cerebellar function.

Chronic alcohol use has been shown to provoke an IgA response that is not only directed towards alcohol metabolites but also against self antigens, targeting the enzyme transglutaminase 2 (TG2), even in the absence of liver disease [9]. Transglutaminases are a widely distributed family of enzymes with cross-linking capability that aid tissue repair [10]. TG2 is the autoantigen in coeliac disease. However, autoimmunity to TG2 can also precipitate hepatitis and ultimately liver failure [11]. Proposed mechanisms for the high prevalence of TG2 autoantibodies in patients with chronic alcohol abuse include increased gut permeability associated with alcohol induced intestinal mucosal lesions [9] leading to increased exposure of the immune system to pathogenic antigens (including gluten peptides). In line with this, alcohol has been shown to induce sensitisation to gluten in genetically susceptible individuals [12].

The aim of this study was to investigate further a potential link between autoreactive immune responses and cerebellar degeneration in the context of excessive alcohol intake.

## Methods

### Subject selection and clinical assessments

The study was approved by the regional ethics committee (Yorkshire & The Humber, UK). Written informed consent was obtained from all patients. Patients were recruited from the Ataxia and Hepatology outpatient clinics at the Royal Hallamshire Hospital, Sheffield, UK. Patients included were grouped as ‘alcohol ataxia (AA)’ and had a history of chronic ‘alcohol dependence’, defined by the 4^th^ edition of the Diagnostic and Statistical Manual of Mental Disorders (DSM-IV) Diagnostic Criteria for Alcohol Abuse and Dependence [13]. A subgroup of patients with alcohol-related ‘chronic liver disease’ (defined as alcohol related liver disease diagnosed by a hepatologist and disease present for more than 1 year) and ataxia (CLDA) was identified from this patient group.

Detailed neurological history and any background history of autoimmune disorders were recorded, as were age at onset, duration of ataxia, requirement for mobility aids, and current alcohol intake or abstinence at the time of recruitment.

Detailed neurological examination was performed. Ataxia was classified as gait, limb or both and severity was assessed as mild (mobilising independently or with one walking aid), moderate (mobilising with 2 walking aids or walking frame) or severe (wheelchair-dependent). The severity assessment was adapted and modified from previously published data [14]. Objective measurement of the severity of ataxia was rated using the Scale for the Assessment and Rating of Ataxia (SARA) [15, 16] (see Additional file 1). All patients were investigated for other causes of ataxia and were excluded if an alternative cause was found. Tests depending on clinical indications included blood cell counts, biochemistry, thyroid function, B12, folate and genetic testing for inherited spinocerebellar ataxias (SCA 1, 2, 3, 6, 7) and Friedreich’s ataxia (FA).

### Brain imaging

Volumetric 3T MR imaging and single-voxel H^1^ MR spectroscopy of the cerebellum were undertaken in patients with clinical evidence of ataxia. This imaging protocol is in clinical use as part of the investigations of all patients with cerebellar ataxia who attend the National Ataxia Centre in the Royal Hallamshire Hospital, Sheffield, UK. The brain imaging protocol for structural, volumetric and spectroscopy studies have been previously reported [12].

The vermis was assessed as a whole for volumetric analysis. A manual approach was adopted for volumetric analysis of the vermis to avoid partial volume averaging effects that may also be affected by the degree of atrophy potentially confounding VBM results (unpublished data). For vermian volumetric studies, the midline vermis was identified on sagittal T1 volume images with reference to the midsagittal plane of the cerebellum [17]. Based on this reference, manual measurement of cross sectional areas of the whole vermis was possible. Vermian volume (V) in mm^3^ was measured as a sum of vermis cross sectional areas (mm^2^) multiplied by 0.9 mm thickness. Vermian volume was expressed as a percentage of the total intracranial volume (%V:TIV).

Healthy controls recruited, were age and gender matched with patient subjects and had undergone the same MR imaging protocol. The details of the healthy controls have also been previously reported [18].

### Blood collection and serological tests

Blood samples were collected at recruitment and the serum was aliquoted to avoid repeat freeze-thawing and stored at −20 ° C in sealed tubes.

All patients had total immunoglobulin levels, IgA and IgG anti-gliadin antibodies (AGA), anti-endomysial antibodies (EMA) and IgA anti-transglutaminase 2 (TG2) antibodies, measured by standard laboratory protocols at the Immunology Department, Northern General Hospital, Sheffield. Briefly, the measurement for AGA and TG2 were performed by enzyme linked immunosorbent assay (ELISA) (Aesku. Diagnostics-Grifols, Germany). EMA was tested by indirect immunofluorescence on slides containing monkey oesophagus tissue (Inova-Instrumentation Laboratories, USA). Patient sera were also used for the detection of IgA and IgG to transglutaminase 6 (TG6) by ELISA as previously described [19].

Human Leukocyte Antigen (HLA) typing was performed at the National Blood Service, Sheffield, UK.

### Immunohistochemistry

Rat cerebellar tissue was acquired from adult Sprague-Dawley rats (Biological Services, Faculty of Medicine, Dentistry & Health, University of Sheffield). The cerebellar tissue was mounted using Cryo-M-Bed tissue embedding polymer (Bright, UK) and snap-frozen with isopentane/cooled on liquid nitrogen. The cerebellar tissue was cryo-sectioned into 10 μm sagittal sections that were collected on polysine-coated slides (Thermo Scientific, UK), stored at −80 ° C in an airtight container.

Mouse anti-calbindin-D-28K monoclonal antibody (Sigma, UK) diluted 1:200 in PBS was used to visualize Purkinje cells. Patient sera were diluted 1:200 or 1:600 in PBS before incubation with sections for 1 h. 1:600 was identified as the optimum dilution in determining the reactivity of patient sera on rat cerebellar tissue in agreement with our previous study [20]. Negative controls included sections incubated without patient sera. A horseradish peroxidase-conjugated goat anti-human or goat anti-mouse IgG antibody (Jackson ImmunoResearch Laboratories, USA) was used as secondary antibodies.

Images were captured at X100 and X200 magnification. Two blinded observers performed the evaluation of the Purkinje cell staining intensity on rat cerebellar sections independently. Staining was classed as ‘weak’ or ‘strong’ if Purkinje cell staining was above background levels. ‘Negative’ was recorded if staining did not exceed background levels. Staining of other neuronal cell populations (granular layer) was also noted. Concordance rate between assessors was 79 %.

### Statistical analysis

Statistical analysis was performed using PRISM 6 software package (GraphPad Software Inc.). Demographic, clinical and imaging characteristics are presented as means with standard deviations (mean ± SD). The Independent-Samples Mann-Whitney U Test was used to determine any difference between mean % cerebellar volume (CBV) : total intracranial volume (TIV) and mean % vermian volume (V) : total intracranial volume (TIV) between patients and controls. The χ^2^ test was used for comparing the prevalence of anti-gliadin antibodies and anti-transglutaminase 6 antibodies in the study group with that of the healthy population; and between the subgroups. Results were considered statistically significant for *p* < 0.05.

## Results

### Clinical presentation

Thirty-eight patients were recruited with mean age of 54 ± 10 years. There were 31 male and 7 female patients. All patients had clinical evidence of ataxia. Thirty of the 38 (79 %) patients were grouped as ‘alcohol ataxia’ (AA) as ataxia was their presenting complaint. A further group of 8/38 (21 %) patients recruited from the Hepatology clinics, were selected on the basis of having alcohol-related chronic liver disease, but were also found to have ataxia, hence categorised as ‘chronic liver disease and ataxia’ (CLDA). Fourteen patients (37 %) had been abstinent from alcohol consumption at the time of recruitment. A background of autoimmune history (diabetes (2), thyroid disorder (1), pernicious anaemia (4)) was seen in 7/38 (18 %) patients.

Age of ataxia onset was 49 ± 11 years. Duration of ataxia ranged from one to 27 years (Mean 6 ± 5 years). Pure gait ataxia was found in 4/38 (11 %) patients and the majority of 34/38 (89 %) patients had both gait and limb ataxia. Nystagmus was present in 11/38 (29 %) patients.

Forty seven percent had mild ataxia (mobilising independently or with one walking aid). Moderate ataxia was seen in 34 % and severe ataxia was seen in 18 % patients. There was no difference in the autoimmune profile between patients with mild, moderate or severe ataxia. The severity of ataxia using the SARA scale revealed a mean total SARA score of 12 ± 7 (range 1 to 26). There was a correlation seen between duration of ataxia and total SARA score (p 0.0272).

Table 1 demonstrates the frequency of involvement for each of the eight key SARA elements. The gait (97 %), stance (89 %) and heel-shin slide (89 %) were the predominant SARA elements affected.

**Table 1:** Scale for the assessment and rating of ataxia (SARA) in patients with alcohol ataxia

SARA elements	AA	CLDA	Total
Gait	29/30 (97 %)	8/8 (100 %)	37/38 (97 %)
Stance	26/30 (87 %)	8/8 (100 %)	34/38 (89 %)
Sitting	5/30 (17 %)	0/8 (0 %)	5/38 (13 %)
Speech disturbance	10/30 (33 %)	1/8 (13 %)	11/38 (29 %)
Finger chase	15/30 (50 %)	5/8 (63 %)	20/38 (53 %)
Nose-finger test	17/30 (57 %)	5/8 (63 %)	22/38 (58 %)
Fast alternating hand movements	18/30 (60 %)	6/8 (75 %)	24/38 (63 %)
Heel-shin slide	27/30 (90 %)	7/8 (88 %)	34/38 (89 %)

### Brain imaging

MR Spectroscopy was performed in 34 patients. Data analysis was based on 32 (25 AA and 7 CLDA) optimal scans. Two scans were excluded as they were of inadequate quality. Abnormal NAA/Cr area ratio (vermis NAA/Cr < 0.95 and/or hemisphere NAA/Cr < 1.00) [21] was recorded in 29/32 (91 %) patients. This included 22/25 (88 %) patients with AA and 7/7 (100 %) patients with CLDA.

Predominantly vermian abnormalities were present in 18/25 (72 %) patients with AA and 6/7 (86 %) patients with CLDA. The hemisphere was solely affected in only 4/25 (16 %) patients with AA and 1/7 (14 %) patients with CLDA.

Volumetric Image analysis matched for age and gender with healthy controls was possible in 28 patients (22 patients with AA and six patients with CLDA). Results are displayed as a whole group (AA and CLDA) vs. controls. Cerebellar volume (%CBV:TIV) was significantly smaller (8.68 ± 0.99) in this study group when compared with age and gender matched healthy controls (9.57 ± 0.96); CI 95 % 8.29 to 9.06, p 0.0027. Similarly, vermian volume (%V:TIV) was significantly smaller in the study group (1.13 ± 0.24) when compared to age and gender matched healthy controls (1.34 ± 0.13); CI 95 % 1.03 to 1.22, p 0.0003.

There was no significant correlation seen between total SARA score and brain imaging measures.

### Serological testing for gluten-related antibodies

Normal total serum immunoglobulins were seen in 21/38 (55 %) patients. Raised IgA levels were seen in 8/38 (21 %) patients.

Circulating anti-gliadin antibodies were detected in 13/38 (34 %) patients (8 AA and 5 CLDA) compared with a 12 % prevalence in healthy controls [22] (χ^2^ p 0.012). The difference between the AA and CLDA groups was not statistically significant. Circulating anti-transglutaminase 2 (TG2) antibodies were demonstrated in 4/38 (11 %) patients, (3 AA and 1 CLDA). The three AA patients had low TG2 antibody titres and the one CLDA patient had TG2 antibody titres >300 U/mL. No patients tested positive for EMA.

Antibodies to transglutaminase 6 (TG6) were detected in 15/38 (39 %) patients vs. 4 % in healthy controls [23] (χ^2^*p* <0.0001). The 15 patients with anti-TG6 antibodies included 9/30 (30 %) patients with AA and 6/8 (75 %) patients with CLDA (χ^2^ p 0.0207). Thirteen of the 15 patients had IgA anti-TG6 antibodies, one had IgG anti-TG6 antibodies and one patient had both IgA and IgG anti-TG6 antibodies.

### HLA genotyping for DQ2/DQ8

Eighteen of the 38 (47 %) patients in the study group had HLA type DQ2 or DQ8. This was not significantly different from the healthy population of 30 % [24] (p 0.056). There was no significant difference between any subgroup (AA vs. CLDA), using the χ^2^ test.

### Serum reactivity with neural tissue

Three specific staining patterns were identified (pure Purkinje cell, pure granular layer and combined Purkinje cell and granular layer staining pattern) when rat brain sections were incubated with patient sera (see Additional file 2). In 27/38 (71 %) patients, one of these patterns was present.

Pure Purkinje cell (PC) staining was demonstrated in 4/38 (11 %) patients. This included 3/30 (10 %) patients with AA and 1/8 (13 %) patients with CLDA. Pure granular layer staining was demonstrated in 16/38 (42 %) patients. This included 12/30 (40 %) patients with AA and 4/8 (50 %) patients with CLDA. Combined Purkinje cell and granular layer staining was demonstrated in 7/38 (18 %) patients that included 5/30 (17 %) patients with AA and 2/8 (25 %) patients with CLDA.

Overall, Purkinje cell reactivity was seen in 11/38 (29 %) of patients and granular layer reactivity in 23/38 (61 %) patients. There was no statistically significant difference in prevalence of Purkinje cell and/or granular layer staining between the 2 subgroups.

Based on the same methodology, our previous studies did not demonstrate any staining in healthy controls and showed only 5 % staining in patients with genetic ataxia [20, 25]. Table 2. summarises the serological characteristics of patients with alcohol ataxia.

**Table 2:** Serological characteristics of patients with alcohol ataxia

Patient ID	Gender	Age at study, y	HLA DQ2/DQ8	Antigliadin IgA and/or IgG	Anti-TG6 IgA and/or IgG	PC stained	GL stained
8	M	33	DQ2		IgA		+
9^a^	M	41					+
20	M	50	DQ8			+	++
27	M	67					++
28	M	56		IgA		+	+
30^a^	M	46					+
31	M	50				++	+
32	M	66			IgA	++	+
33	M	58	DQ8				
37	M	53	DQ2	IgA			+
38	F	56		IgA			+
45	M	49	DQ8				+
47	F	60		IgA			
49^a^	M	51	DQ8	IgA, IgG	IgA		
55	F	48	DQ2			+	
62	M	41					
69	M	58					
92	M	64	DQ8			++	+
100	M	55	DQ2		IgA		
101	F	49	DQ2				+
103	F	48	DQ8	IgA			+
104	M	55	DQ2				+
107^a^	M	46	DQ2	IgA	IgA	++	++
116	M	51	DQ8		IgG		++
119	M	48			IgA		++
120^a^	M	50			IgA		+
121	M	45					+
122^a^	M	50		IgA	IgA		+
125	F	66	DQ2		IgA		
128	M	68	DQ2		IgA		
132	M	62					
133^a^	M	59	DQ2	IgA	IgA	+	++
134	F	58				+	
135	M	57		IgA	IgA	+	
136^a^	M	47		IgA	IgA	+	
139	M	65	DQ2	IgA, IgG	IgA, IgG		
143	M	67		IgG			
145	M	81					+

^a^subgroup chronic liver disease and ataxia (CLDA), *y* years, *TG6* transglutaminase 6, *PC* Purkinje cell layer, *GL* granular layer, *+* weak staining, *++* strong staining

## Discussion

In this study, we investigated whether an autoimmune process may drive alcohol-induced cerebellar damage. Our initial intention was to study patients with alcoholic liver disease without ataxia and a subgroup of patients who presented with alcohol-induced ataxia. However, all patients with alcoholic liver disease were found to also have ataxia thus a subgroup of patients with ‘chronic liver disease and ataxia’ was defined. This division into 2 subgroups is somewhat arbitrary as patients with alcohol-induced ataxia almost always have some degree of liver involvement (demonstrated by elevated levels of gamma GT). Given the overlap in functional deficits, our sample size would also be too small to make any definitive conclusions on specific differences between the 2 subgroups. Nonetheless, we still studied the 2 subgroups given the distinct differences in presentation.

Imaging data using MRI volumetric and spectroscopy techniques demonstrated significant structural and functional deficits of the cerebellum, with preferential involvement of the cerebellar vermis. There was significant vermian volume loss in patients with alcohol ataxia compared to age and gender matched healthy controls. The findings support neuropathological data that alcohol-related cerebellar degeneration preferentially affects the cerebellar vermis [2–7]. Vermian involvement is common in immune-mediated ataxias such as gluten ataxia, paraneoplastic cerebellar degeneration and primary autoimmune cerebellar ataxia [26]. We did not find any correlation between the imaging findings and immunohistochemical findings in this cohort. The sample size, however, may be a contributory factor to this.

Alcohol excess is associated with impairment of the blood-brain barrier. The oxidative stress caused by alcohol metabolism on brain microvascular endothelial cells by activation of myosin light chain kinase leads to disruption of the tight junctions inducing blood-brain barrier breakdown [27, 28]. This enhanced permeability may lead to neo-epitope exposure to the immune system and thereby induction of autoimmune responses to these neo-epitopes or allow reaction of serum antibodies with neural tissue and consequently trigger localized inflammatory processes in the brain. In line with this, we show that 71 % of patients with alcohol ataxia have circulating antibodies that react with neural tissue. Three cerebellar staining patterns were seen when rat brain sections were incubated with patient sera: pure Purkinje cell (11 %), pure granular layer (42 %) and combined Purkinje cell and granular layer (18 %). We have previously shown that up to 60 % of patients with idiopathic sporadic ataxia have anti-cerebellar antibodies as opposed to 5 % of patients with genetic ataxia [25]. It is possible that alcohol is a contributing factor to “idiopathic” sporadic ataxia, or that similar immune-mediated processes can be triggered by infection or other unrelated environmental factors in addition to alcohol.

Increased gut permeability and mucosal damage are typical clinical findings in patients with alcohol misuse. Not surprisingly, this may trigger a breakdown in immune tolerance and autoimmune responses in patients harbouring genetic susceptibility (HLA DQ2/DQ8). Excessive alcohol intake is a risk factor for coeliac disease development [9, 12]. TG2 IgA autoantibody titres have been reported to positively correlate with liver disease [9] which is in accordance with the view that liver disease *per se* may be associated with the occurrence of TG2 autoantibodies [11]. The role of TG in tissue repair and its deposition into the extracellular matrix [29] may lead to abundant autoantibody binding and thereby drive chronic inflammation and fibrogenesis. Only one of the patients examined here had high levels of circulating anti-TG2 autoantibodies suggesting that coeliac disease is not a primary underlying mechanism in this cohort selected on the basis of neurological deficits. However, in gluten-related disorders with extraintestinal manifestation, particularly neurological, enteropathy may not be present and autoantibodies to TG2 may not be detectable in the circulation [19, 23].

It is therefore of interest that firstly, we found a significantly higher prevalence of anti-gliadin antibodies in the study group (34 %) compared to healthy controls (12 %) and secondly that we found a significantly higher prevalence of anti-TG6 antibodies in the study group (39 %) compared to healthy control subjects (4 %). Circulating anti-TG6 antibodies are the best marker currently available for patients with primarily neurological presentation of gluten-related disorders [23]. However, their prevalence in the study group was not substantially different to what we previously reported for idiopathic ataxia (32 %), and the association of AGA and anti-TG6 autoantibodies is in line with previous findings (gluten ataxia) [23]. Clearly, these antibodies alone cannot explain the serum reactivity to the granular layer neurons in most of these patients [20, 30]. Anti-TG6 antibodies were more prevalent in the subgroup of patients with CLDA (75 %) compared to the subgroup of patients with AA (30 %) suggesting that alcohol-related chronic liver disease itself may be another risk factor for the development of autoimmunity to TG6. It remains to be shown where the immune response to TG6 develops. The fact that almost exclusively an IgA response was seen makes the gastrointestinal tract a likely candidate.

## Conclusions

Taken together, these results suggest that factors other than direct cellular toxicity from alcohol, including autoimmune responses, may play a part in cerebellar degeneration in patients with alcohol ataxia. The role of gluten sensitivity merits further exploration but the findings of this and previous studies suggest that sensitivity to gluten may be an epiphenomenon, likely resulting from increased gut permeability. Development of gluten-related autoantibodies, however, may perpetuate cerebellar degeneration even when the patient abstains from alcohol intake. If that proves to be the case, a gluten free diet may prove to be beneficial for such patients.

## Additional files

Additional file 1: Scale for the Assessment and Rating of Ataxia (SARA). (PDF 33 kb) Additional file 2: Immunohistochemistry staining patterns illustrating serum reactivity with neural tissue. (PDF 2951 kb)

## Abbreviations

AGA: Anti-gliadin antibody
CNS: Central nervous system
EMA: Endomysial antibody
HLA: Human leukocyte antigen
MRI: Magnetic resonance imaging
NAA/Cr: N-acetyl aspartate:creatine ratio
SARA: Scale for the assessment and rating of ataxia
TG2: Transglutaminase 2
TG6: Transglutaminase 6
