# Supplementary material for: Alcohol-related cerebellar degeneration: not all down to toxicity?
Source: Cerebellum Ataxias. 2016 Oct 3;3:17. doi: 10.1186/s40673-016-0055-1 (PMC5048453; doi:10.1186/s40673-016-0055-1)
Supplement: Additional file 1: — Scale for the Assessment and Rating of Ataxia (SARA). (PDF 33 kb) [file 40673_2016_55_MOESM1_ESM.pdf]

Rater: ____________________________date: ______________patient:________________________

Scale for the assessment and rating of ataxia (SARA)

1) Gait

Proband is asked (1) to walk at a safe distance parallel to a wall including a half-turn (turn around to face the opposite direction of gait) and (2) to walk in tandem (heels to toes) without support.

0	**Normal, no difficulties in walking, turning and walking tandem (up to one misstep allowed)**
1	**Slight difficulties, only visible when walking 10 consecutive steps in tandem**
2	**Clearly abnormal, tandem walking >10 steps not possible**
3	**Considerable staggering, difficulties in half-turn, but without support**
4	**Marked staggering, intermittent support of the wall required**
5	**Severe staggering, permanent support of one stick or light support by one arm required**
6	**Walking > 10 m only with strong support (two special sticks or stroller or accompanying person)**
7	**Walking < 10 m only with strong support (two special sticks or stroller or accompanying person)**
8	**Unable to walk, even supported**

Score	

2) Stance

Proband is asked to stand (1) in natural position, (2) with feet together in parallel (big toes touching each other) and (3) in tandem (both feet on one line, no space between heel and toe). Proband does not wear shoes, eyes are open. For each condition, three trials are allowed. Best trial is rated.

0	**Normal, able to stand in tandem for > 10 s**
1	**Able to stand with feet together without sway, but not in tandem for > 10s**
2	**Able to stand with feet together for > 10 s, but only with sway**
3	**Able to stand for > 10 s without support in natural position, but not with feet together**
4	**Able to stand for >10 s in natural position only with intermittent support**
5	**Able to stand >10 s in natural position only with constant support of one arm**
6	**Unable to stand for >10 s even with constant support of one arm**

Score	

3) Sitting

Proband is asked to sit on an examination bed without support of feet, eyes open and arms outstretched to the front.

0	**Normal, no difficulties sitting >10 sec**
1	**Slight difficulties, intermittent sway**
2	**Constant sway, but able to sit > 10 s without support**
3	**Able to sit for > 10 s only with intermittent support**
4	**Unable to sit for >10 s without continuous support**

Score	

4) Speech disturbance

Speech is assessed during normal conversation.

0	**Normal**
1	**Suggestion of speech disturbance**
2	**Impaired speech, but easy to understand**
3	**Occasional words difficult to understand**
4	**Many words difficult to understand**
5	**Only single words understandable**
6	**Speech unintelligible / anarthria**

Score	

Rater: ____________________________date: ______________patient:________________________

5) Finger chase

Rated separately for each side
Proband sits comfortably. If necessary, support of feet and trunk is allowed. Examiner sits in front of proband and performs 5 consecutive sudden and fast pointing movements in unpredictable directions in a frontal plane, at about 50 % of proband´s reach. Movements have an amplitude of 30 cm and a frequency of 1 movement every 2 s. Proband is asked to follow the movements with his index finger, as fast and precisely as possible. Average performance of last 3 movements is rated.

0 No dysmetria
1 Dysmetria, under/ overshooting target <5 cm
2 Dysmetria, under/ overshooting target < 15 cm
3 Dysmetria, under/ overshooting target > 15 cm
4 Unable to perform 5 pointing movements

Score	Right	Left
mean of both sides (R+L)/2		

6) Nose-finger test

Rated separately for each side
Proband sits comfortably. If necessary, support of feet and trunk is allowed. Proband is asked to point repeatedly with his index finger from his nose to examiner’s finger which is in front of the proband at about 90 % of proband’s reach. Movements are performed at moderate speed. Average performance of movements is rated according to the amplitude of the kinetic tremor.

0 No tremor
1 Tremor with an amplitude < 2 cm
2 Tremor with an amplitude < 5 cm
3 Tremor with an amplitude > 5 cm
4 Unable to perform 5 pointing movements

Score	Right	Left
mean of both sides (R+L)/2		

7) Fast alternating hand movements

Rated separately for each side
Proband sits comfortably. If necessary, support of feet and trunk is allowed. Proband is asked to perform 10 cycles of repetitive alternation of pro- and supinations of the hand on his/her thigh as fast and as precise as possible. Movement is demonstrated by examiner at a speed of approx. 10 cycles within 7 s. Exact times for movement execution have to be taken.

0 Normal, no irregularities (performs <10s)
1 Slightly irregular (performs <10s)
2 Clearly irregular, single movements difficult to distinguish or relevant interruptions, but performs <10s
3 Very irregular, single movements difficult to distinguish or relevant interruptions, performs >10s
4 Unable to complete 10 cycles

Score	Right	Left
mean of both sides (R+L)/2		

8) Heel-shin slide

Rated separately for each side
Proband lies on examination bed, without sight of his legs. Proband is asked to lift one leg, point with the heel to the opposite knee, slide down along the shin to the ankle, and lay the leg back on the examination bed. The task is performed 3 times. Slide-down movements should be performed within 1 s. If proband slides down without contact to shin in all three trials, rate 4.

0 Normal
1 Slightly abnormal, contact to shin maintained
2 Clearly abnormal, goes off shin up to 3 times during 3 cycles
3 Severely abnormal, goes off shin 4 or more times during 3 cycles
4 Unable to perform the task

Score	Right	Left
mean of both sides (R+L) / 2		
