# Supplementary material for: Alcohol-related cerebellar degeneration: not all down to toxicity?
Source: Cerebellum Ataxias. 2016 Oct 3;3:17. doi: 10.1186/s40673-016-0055-1 (PMC5048453; doi:10.1186/s40673-016-0055-1)
Supplement: Additional file 2: — Immunohistochemistry staining patterns illustrating serum reactivity with neural tissue. (PDF 2951 kb) [file 40673_2016_55_MOESM2_ESM.pdf]

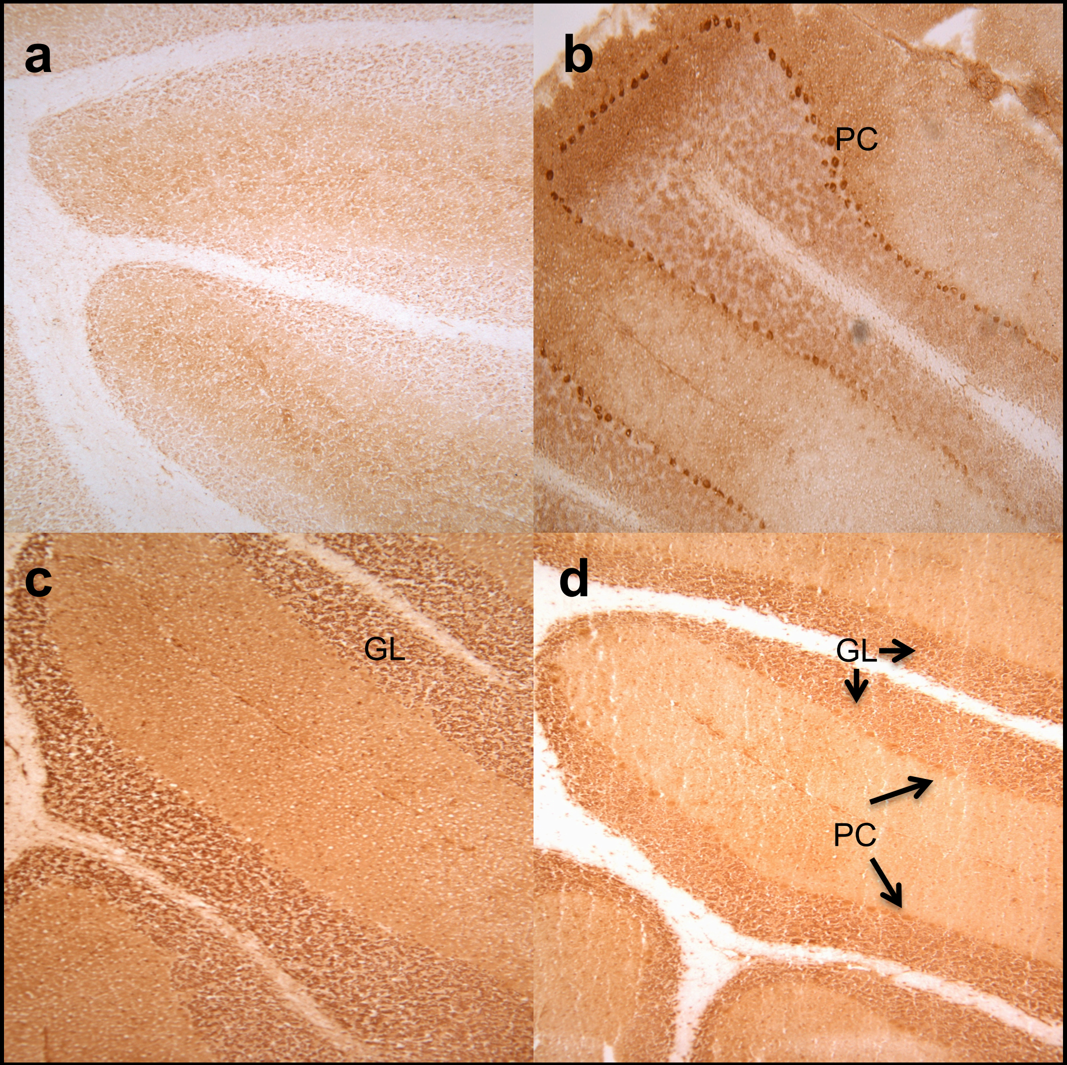

Immunohistochemistry staining patterns (1:600 dilution, x100 magnification) illustrating serum reactivity with neural tissue. a: sera control, b: Purkinje cell (PC) predominant stain, c: granular layer (GL), d: combined Purkinje cell (PC) and granular layer (GL)
